# Granulomatous Mastitis: A Rare Case with Sjogren’s Syndrome and Complications

**DOI:** 10.7759/cureus.5359

**Published:** 2019-08-10

**Authors:** George Yazigi, Becca H Trieu, Michael Landis, Jignesh G Parikh, Mamta Mangal

**Affiliations:** 1 Internal Medicine, University of Central Florida College of Medicine, Orlando, USA; 2 Miscellaneous, University of Central Florida College of Medicine, Orlando, USA; 3 Pathology, Orlando VA Medical Center, Orlando, USA; 4 Internal Medicine, University of Central Florida College of Medicine/Orlando VA Medical Center, Orlando, USA

**Keywords:** granulomatous mastitis, sjogren's syndrome, mastitis

## Abstract

Granulomatous mastitis (GM) is a rare benign chronic inflammatory process of the breast in reproductive aged females. Although considered idiopathic in many cases, it has been associated with other conditions. Herein we report a highly complex and interesting case of GM in a young female with Sjogren's syndrome. We also review the literature and discuss challenges pertaining to the management of patients with similar risk factors. According to our knowledge, this is the third case documenting the co-occurrence of GM and Sjogren's syndrome. We focus on the challenges and complications of GM in the context of an autoimmune disease. With evidence from our patient’s disease course and support from the literature, we advocate the use of corticosteroids for GM to prevent complications in patients with additional risks factors such as an autoimmune disease.

## Introduction

Idiopathic granulomatous mastitis (IGM) is a rare benign chronic inflammatory process of the breast with an unknown and debatable etiology [1]. First discussed in the literature in 1972 by Kessler and Wolloch, IGM was originally described as having a pathogenesis revolved around an autoimmune process [2]. IGM typically presents as a unilateral painful breast mass in a reproductive aged female, with a median age of diagnosis in the mid-30s [1,3-4]. Other possible presenting findings may include nipple discharge, nipple retraction, fistula formation, and skin hyperemia [1,5]. If there is a suspected cause of disease, IGM would be called granulomatous mastitis (GM). Both IGM and GM present similarly to other more concerning conditions such as carcinoma and infection [1,5]. Clinical findings and imaging for GM are nonspecific and overlap with other diagnoses, which could delay the treatment [5]. A definitive diagnosis of IGM requires a biopsy with histopathologic evidence of inflammatory infiltrates and epithelioid histiocytes defining noncaseating granulomas [6]. Even with a biopsy of noncaseating granulomatous inflammation, a group of potential causes must be excluded to make a diagnosis of IGM versus GM. Some of these may include well-known causes of granulomas like sarcoidosis and Mycobacterium tuberculosis infection [7]. This becomes significant as some believe the high amount of reports of IGM diagnoses from developing countries could be a misdiagnosis of GM due to tuberculosis.

Given the low prevalence of GM, there has not been a development for a consistent treatment guideline [3]. Treatments that have shown efficacy include observation, antibiotics, excision, and corticosteroids [3-4]. The difficulty in treating GM could potentially lead to complications such as fistula formation, abscesses, post-procedural infections, and recurrences [7]. In our case, we will be discussing the complications subsequent to a diagnosis of GM in a patient with existing Sjogren’s syndrome which was hypothesized to be associated with GM but is underreported in the literature.

## Case presentation

**Figure 1:**
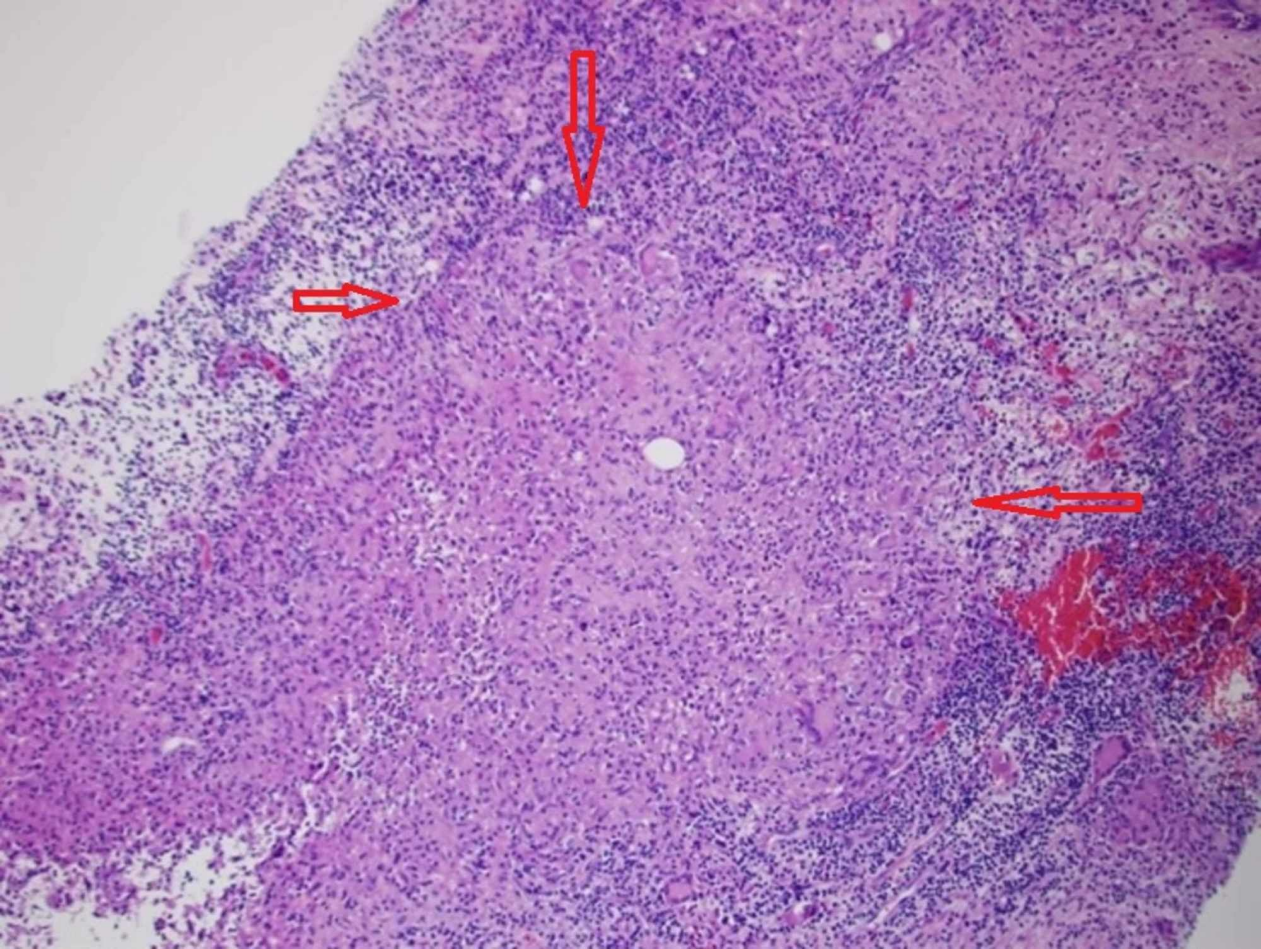
Breast biopsy Breast biopsy shows noncaseating granulomatous inflammation, consistent with granulomatous mastitis (between red arrows).

The patient is a 37-year-old Hispanic female who initially presented for a firm mass on her right breast that had enlarged and became painful over one year. Her medical history is significant for Sjogren’s syndrome with no systemic treatment, hypothyroidism, parity, a right breast benign adenoma, and bilateral breast augmentation with subsequent removal of the implants due to infection. A fine-needle aspiration (FNA) biopsy showed GM (Figure 1). Right axillary lymph node contained lymphoid tissue with sinus histiocytosis. There was no evidence of cancer, fungus, or tuberculosis. A diagnosis of idiopathic GM was made. However, no treatment was given as she was lost to follow-up.

**Figure 2:**
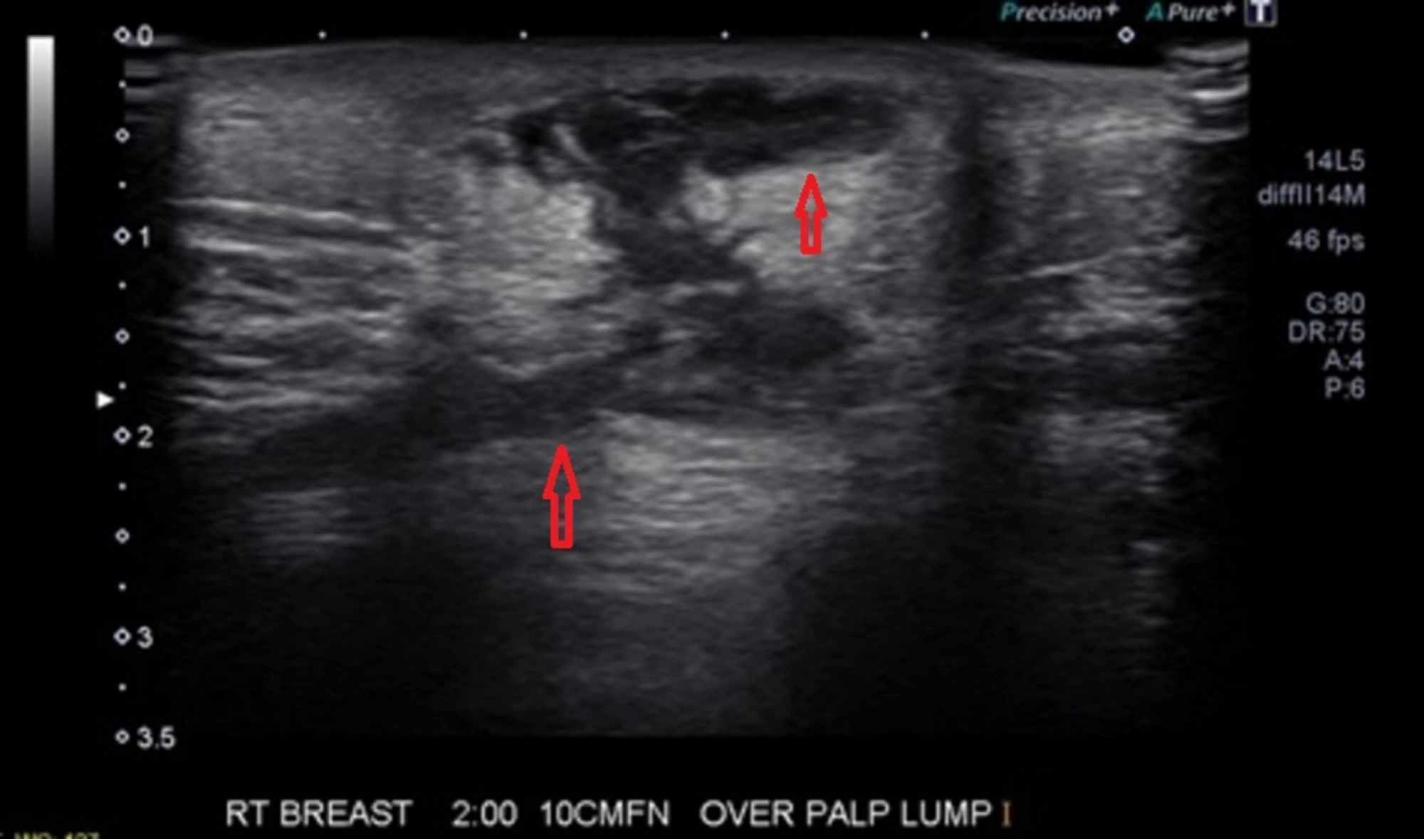
Breast ultrasound Breast ultrasound (US) before incision and drainage (I&D) shows complex partially fluid-filled parenchymal changes directly under the indurated area (upper red arrow) with extension into the biopsied granulomatous mastitis lesion suspicious for fistularization with possible abscess or phlegmon formation (lower red arrow).

Three months after the FNA, her incision site did not fully heal while the mass continued to grow. There was worsening erythema, induration, and pain associated with the mass. Ultrasound (US) of the mass showed complex partially fluid-filled parenchymal changes directly under the indurated area with extension into the biopsied GM lesion suspicious for fistularization with possible abscess or phlegmon formation (Figure 2). US findings did not indicate that the mass was drainable. Although her labs did not indicate an active infection, the patient was placed on oral linezolid 400 mg twice daily for 14 days and asked to follow-up in the outpatient surgical clinic.

**Figure 3:**
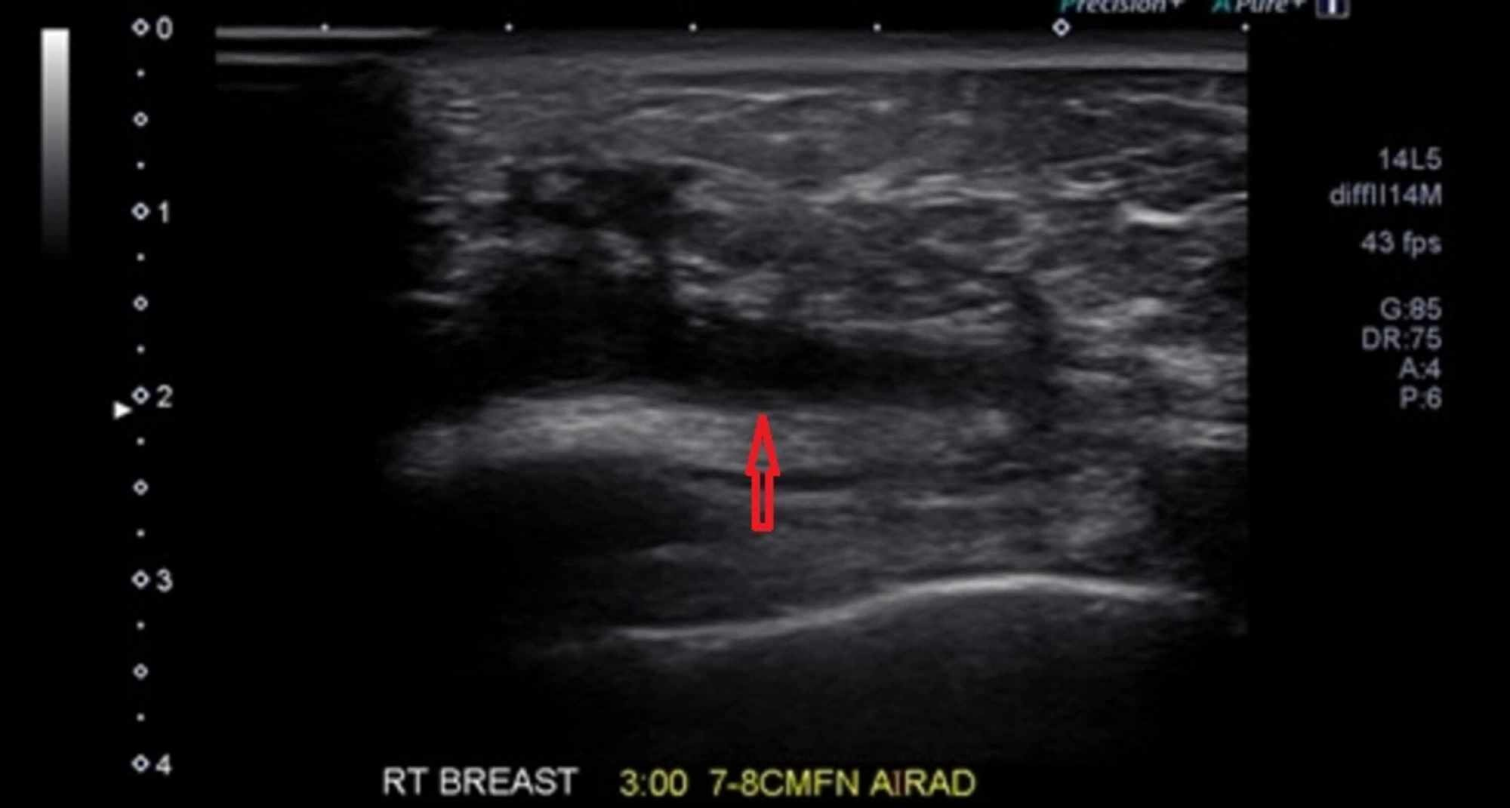
Breast ultrasound Breast ultrasound (US) post incision and drainage (I&D) shows resolution of parenchymal fluid and confirmation of a persistent path between the I&D site and the biopsied granulomatous mastitis lesion (red arrow).

Prior to the completion of her antibiotic course, she presented with thick drainage from the site of the mass. This presentation was consistent with a breast abscess, for which the patient underwent an incision and drainage (I&D) with packing. During the post-op evaluation, the site of I&D showed granulation and sanguineous drainage without any signs of infection which was supported by her labs and vital signs. Post-I&D US showed resolution of parenchymal fluid and confirmation of a persistent path between the I&D site and the biopsied GM lesion (Figure 3). Differential diagnoses from US included progression of GM with fistularization and a superimposed infection with more evidence for the former diagnosis. One notable drawback during the investigation for her case is the lack of a bacterial culture; hence, we could not definitively rule out infection as a complication associated with her GM. A week after I&D, the patient reported some improvement in the I&D site.

## Discussion

IGM is a poorly understood condition with only a few hundred cases reported worldwide [8]. IGM has been suggested to have a 1.8% incidence among females presenting with breast pathology [5]. Many different risk factors have been proposed for the development of IGM. Prior pregnancy, autoimmune conditions, infection, hyperprolactinemia, and oral contraceptives have all been proposed as potential contributing factors [9-10]. There is a higher incidence in Hispanic women, as was our patient [4]. Autoimmunity has been viewed as a main cause of GM due to the favorable response of the condition to corticosteroids [2]. Additionally, certain other risk factors such as pregnancy, smoking, abscess formation, and lactation have been discussed as increasing the risk for recurrence [3,11]. One study has shown up to a 50% recurrence rate [12]. However, amongst these factors, the etiology of autoimmune versus infectious is still debatable due to a lack of consistent findings [7,13-14]. 

Our patient presented with risk factors that historically have been considered related to the development of GM. These factors include her age, race, history of pregnancy, Sjogren’s syndrome, and a potentially autoimmune hypothyroidism. Sjogren’s syndrome is an autoimmune disease that affects exocrine glands, mainly leading to infiltration of the lacrimal and salivary glands. Although it has been hypothesized that GM is associated with other autoimmune conditions, one of which is autoimmune thyroiditis, there is little solid evidence for its association with Sjogren’s syndrome [2,15-16]. As Sjogren’s affects exocrine glands, it is also possible for mammary glands to be affected. Our case further reinforces the rare finding of a possible association with Sjogren’s. Her Sjogren’s was diagnosed by an ophthalmologist who managed it solely with dry-eye treatments. No treatment with steroids was given after biopsy showed GM since she was lost to follow-up.

Due to the low incidence and lack of standardized treatment guidelines for GM, many physicians are unfamiliar with treating this condition. A case series with only nine cases has shown that 50% of patients with IGM spontaneously resolved breast masses without any treatments [6]. Although close observation is a possible approach for GM, a lack of treatment may have contributed to our patient’s complex course of disease. Literature has shown that trauma such as an FNA can increase the risk for complications [7,17]. Looking at our patient’s case retrospectively, we believe steroid therapy may have prevented her abscess and fistula.

Abscess and fistula development are possible sequelae of GM [10]. Our patient was found to have a fistula with possible abscess on ultrasound following an FNA that was done three months prior. In a 2006 retrospective chart review, only one of the 21 cases of GM presented with fistula formation [14]. A study in Turkey of 63 patients found 45.5% with abscess formation [18]. GM has no optimal treatment, and the development of abscesses and/or fistula make the disease even more difficult to treat as wide local excision may not be successful and could be disfiguring [4,19]. A compounding difficulty related to fistula and abscesses is indicated by research showing that they are predictors for recurrences [18]. A study in Jordan shows successful conservative treatment of fistulae in IGM with medications instead of surgery [20]. This compilation of evidence further reinforces our preference for corticosteroids in cases like our patient’s.

## Conclusions

This case offers more support for the rare-documented association between Sjogren’s syndrome and GM. To our knowledge, this is the third instance to show such an association. In our patient’s case, the word “idiopathic” should be retracted when Sjogren's syndrome is the suspected cause of her GM. This paper advocates the use of corticosteroids in a subpopulation of patients with GM who have risk factors resembling the case presented. As an autoimmune condition likely caused and attenuated our patient’s GM, she was likely placed at a higher risk of complications when enduring trauma via FNA without subsequent steroid therapy. To prevent these complications, patients with similar risk factors should be placed on corticosteroids right after their FNA result shows GM. Once developed, abscess and fistula formation complicate the management and surgical excision of GM. It conveys worsened prognosis with higher recurrence rates. The complications may respond well to corticosteroid treatments which further supports our recommendation for their use. Further research, especially randomized-control studies with a larger sample size, should be conducted to explore optimal treatments for patients with different risk profiles.
